# Implementing a flipped classroom model in an evidence-based medicine curriculum for pre-clinical medical students: evaluating learning effectiveness through prospective propensity score-matched cohorts

**DOI:** 10.1186/s12909-022-03230-z

**Published:** 2022-03-16

**Authors:** Yen-Po Tsao, Wan-Yu Yeh, Teh-Fu Hsu, Lok-Hi Chow, Wei-Chih Chen, Ying-Ying Yang, Boaz Shulruf, Chen-Huan Chen, Hao-Min Cheng

**Affiliations:** 1 Division of Allergy, Immunology, and Rheumatology, Department of Medicine, Taipei Veterans General Hospital, Taipei, Taiwan; 2 Institute of Clinical Medicine, National Yang Ming Chiao Tung University College of Medicine, Taipei, Taiwan; 3 Center for Evidence-based Medicine, Taipei Veterans General Hospital, No. 201, Sec. 2, Shih-Pai Road, Taipei, Taiwan; 4 Department of Emergency Medicine, Taipei Veterans General Hospital, Taipei, Taiwan; 5 Institute of Emergency and Critical Care Medicine, National Yang Ming Chiao Tung University College of Medicine, Taipei, Taiwan; 6 Department of Anesthesiology, Taipei Veterans General Hospital, Taipei, Taiwan; 7 Taiwan Joanna Briggs Institute Collaborating Center, National Yang Ming Chiao Tung University, Taipei, Taiwan; 8 Department of Medical Education, Taipei Veterans General Hospital, Taipei, Taiwan; 9 Department of Chest Medicine, Taipei Veterans General Hospital, Taipei, Taiwan; 10 Department of Medicine, National Yang Ming Chiao Tung University College of Medicine, Taipei, Taiwan; 11 Office of Medical Education, University of New South Wales, Sydney, Australia; 12 Institute of Public Health, National Yang Ming Chiao Tung University College of Medicine, Taipei, Taiwan

**Keywords:** Undergraduate medical education, Curriculum reform, Efficacy of educational programs, Evidence-based medicine (EBM), Flipped classroom

## Abstract

**Background:**

In a flipped classroom (FC) model, blended learning is used to increase student engagement and learning by having students finish their readings at home and work on problem-solving with tutors during class time. Evidence-based medicine (EBM) integrates clinical experience and patient values with the best evidence-based research to inform clinical decisions. To implement a FC and EBM, students require sufficient information acquisition and problem-solving skills. Therefore, a FC is regarded as an excellent teaching model for tutoring EBM skills. However, the effectiveness of a FC for teaching EBM competency has not been rigorously investigated in pre-clinical educational programs. In this study, we used an innovative FC model in a pre-clinical EBM teaching program.

**Methods:**

FC’s teaching was compared with a traditional teaching model by using an assessment framework of prospective propensity score matching, which reduced the potential difference in basic characteristics between the two groups of students on 1:1 ratio. For the outcome assessments of EBM competency, we used an analysis of covariance and multivariate linear regression analysis to investigate comparative effectiveness between the two teaching models. A total of 90 students were prospectively enrolled and assigned to the experimental or control group using 1:1 propensity matching.

**Results:**

Compared with traditional teaching methods, the FC model was associated with better learning outcomes for the EBM competency categories of Ask, Acquire, Appraise, and Apply for both written and oral tests at the end of the course (all *p*-values< 0.001). In particular, the “appraise” skill for the written test (6.87 ± 2.20) vs. (1.47 ± 1.74), *p* < 0.001), and the “apply” skill for the oral test (7.34 ± 0.80 vs. 3.97 ± 1.24, *p* < 0.001) had the biggest difference between the two groups.

**Conclusions:**

After adjusting for a number of potential confunding factors, our study findings support the effectiveness of applying an FC teaching model to cultivate medical students’ EBM literacy.

**Supplementary Information:**

The online version contains supplementary material available at 10.1186/s12909-022-03230-z.

## Background

Flipped classroom (FC) teaching is an innovative instructional strategy designed to increase student engagement and learning by having students finish their readings at home and work on problem-solving with tutors during class time [1]. It is a contrast to the traditional teacher-centered instructional approach. In a FC, basic knowledge is self-taught before the class, using materials provided by the teacher, such as videos or other non-traditional learning materials. Then, the teacher guides the students through peer collaboration to strengthen what they have learned on their own, through practical problem solving. The spirit of a FC is to allow teachers to truly engage in two-way communication teaching activities in a face-to-face classroom learning environment, helping students not only acquire skills obtained in a traditional classroom (e.g., memorization, comprehension), but also to achieve higher-level learning capabilities, such as to creating, applying, analyzing, or evaluating information. A FC’s instructional design is supported by the constructivist learning theory [2–5]. Previous studies have suggested the advantages of applying FC include: the improvement of students’ learning autonomy, the easier discovery of blind spots in students’ learning through students’ demonstration of pre-class reading, the more flexible presentation of teaching materials to encourage students’ classroom participation, the encouragement of students’ cooperation inside and outside the class, more efficient use of classroom time, etc. [6–9]. With the rapid development of information technology pushing up the trend of digital transformation of educational activities, FC has received great attention from various educational fields [5, 7], the FC model has also been increasingly applied to the field of clinical teaching in recent years, including medical, pharmacy, nursing, and other health science fields [10]. With the introduction of teaching concepts and technical resources, the FC model providing teachers with more choices in the design of teaching activities, for example, encourage independent learning, gamified learning, etc. [8, 9].

From the research literature and teaching practice experience [11, 12], we believe that the application of the FC model to evidence-based medicine (EBM) teaching may have considerable potential. The FC model seems fit well with the expectations of prelicensure medical courses, in which students became motivative, self-directed learners have confidence in critical thinking and clinical decision-making. However, to date, there are still limited applications of the FC model for rigorously evaluating learning effectiveness in evidence-based medicine (EBM) classes.

EBM mainly uses epidemiological and statistical methods to identify trustworthy evidence within the larger medical database, through rigorous evaluation and comprehensive appraisal. The best evidence-based literature is then combined with the professional experience of clinicians and the values and expectations of patients, which is then integrated and applied to clinical services, so that patients can receive the best care [13]. Improved EBM competency is required to enrich the clinical profession and enhance understanding of patient situations. This includes recognizing the knowledge gaps in textbooks, emphasizing individual responsibility in pursuing knowledge, and turning knowledge gaps into opportunities for problem-solving. Through systematic data collection, and compilation and analysis of evidence, clinical staff are encouraged to apply evidence-based decisions in daily clinical work [14, 15]. Therefore, integrating EBM into teaching requires comprehensive improvement in students’ cognition, attitude, and behavior. This is not only relevant to medical classroom education, but is also a necessary skill for lifelong personal learning. The clinical competencies of EBM have been listed as one of the core abilities of medical staff by the Institute of Medicine (IOM) and the Accreditation Council for Graduate Medical Education (ACGME) in the US. Our previous study demonstrated that using clinical scenarios in teaching EBM principles can result in better learning outcomes than conventional didactic lectures [16]. To implement a FC and EBM, students require sufficient information acquisition and problem-solving skills. Therefore, a FC is regarded as an excellent teaching model for tutoring EBM skills. Past literatures have also pointed out the elements required for the successful application of the FC model to develop innovative teaching [7, 17], such as: (1) institutional support of educational institutions; (2) teachers’ ability to integrate teaching media and technology; (3) teachers’ belief in guiding students to learn independently; (4) flexible use of real-world teaching strategies in classrooms. However, the specific demonstration of teaching effectiveness is more helpful to promote the development of these favorable FC factors.

To confirm the effects of a novel strategic curriculum design, a rigorous curriculum evaluation is required for presenting empirical evidence of student learning outcomes. However, randomized control trials are not usually feasible in real-world educational environments, in which many confounding factors might bias the final study results. Until now, evaluating strategic effectiveness remains a challenge to medical education practitioners. Previous research emphasizes that the lack of strategic curriculum evaluation methods has caused a bottleneck in promoting medical education reform and implementing innovative curricula [14].

Prospective propensity score matching (PSM) has received increasing attention because it can more reasonably evaluate an intervention’s effectiveness by reducing the influence of selection bias and confounding variables commonly observed in observational studies. Through the PSM method, the probability value of each case assigned to the experimental group, the propensity score, can be used when selecting for the control group, which subsequently helps match the attributes of the participants between the experimental and control group, and establish a causal relationship between the experimental treatment and the results [18, 19]. The present study applied this rigorous teaching evaluation framework, referred to as the prospective propensity score matching assessment (PPSMA), to investigate the effectiveness of this innovative EBM course.

Through the use of PPSMA in assessing different domains of EBM skills, including how to ask for, acquire, appraise, and apply the newly obtained knowledge [15], the present study aimed to compare learning outcomes between two prospectively enrolled study cohorts participating in either the experimental group (FC teaching) or the control group, that is, traditional lecture-based (LB) teaching.

## Methods

### Participants and study design

**Fig. 1:**
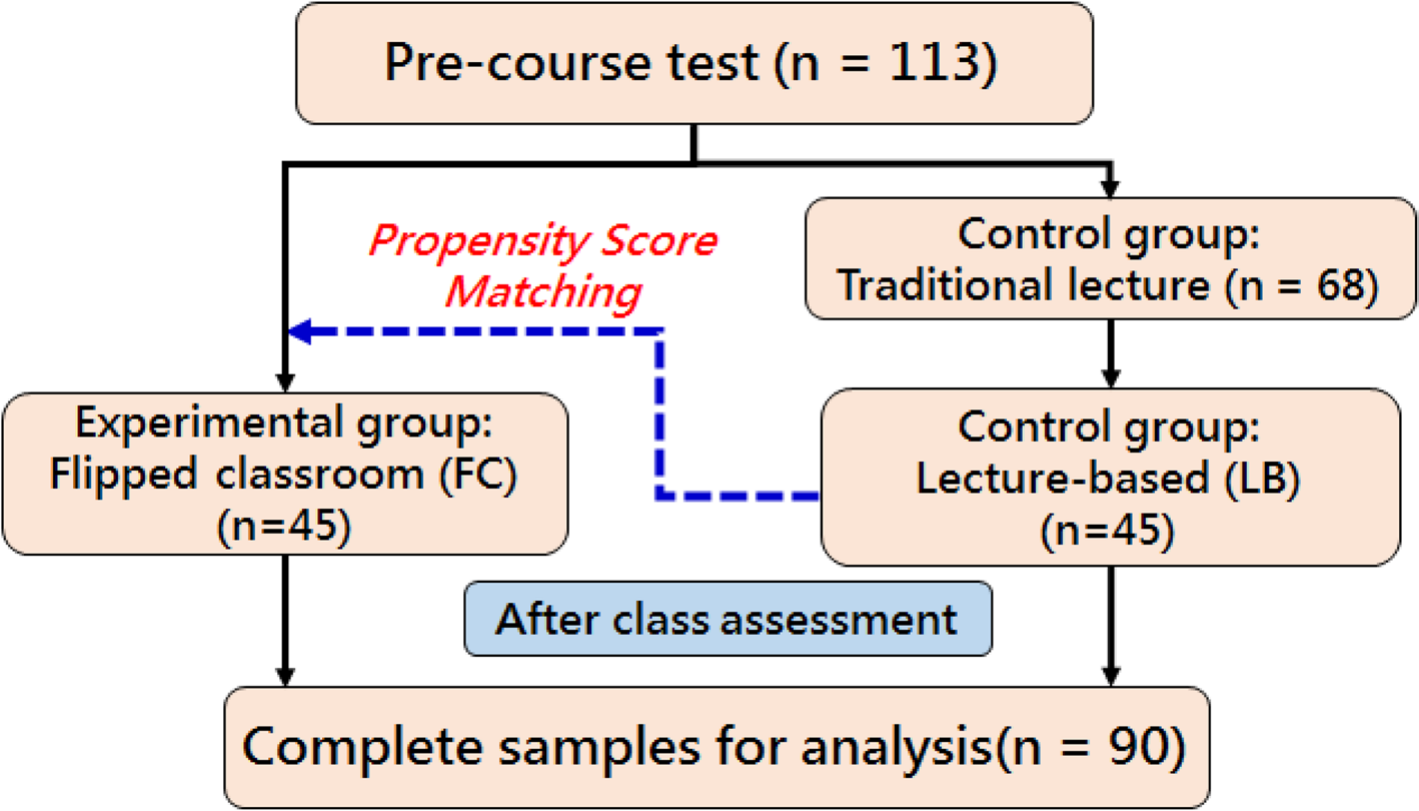
Research Flowchart

Study participants were recruited from October 2016 to August 2018, which included three semesters. The FC group was composed of fifth-year students from the Department of Medicine of Yang Ming Chiao Tung University, who received clinical clerkship training in an elective evidence-based medicine (EBM) skills course. (Since 2013, Taiwan’s medical education system has been changed to a six-year system, and clinical medical training is carried out in the fifth and sixth years. The students participating in this study were enrolled in their basic clinical clerkships.) During the course, students would review course video clips and read the case discussion materials before class. Then, during class, the teacher would enhance the students’ basic EBM skills training by including how to address clinical problems, how to search, evaluate, and apply relevant literature, and how to use these abilities during clinical care through clinical case discussions. The LB group consisted of medical students who completed a clerkship in general internal medicine at Taipei Veterans General Hospital during the same study period. In their general internal medicine clinical training course, there is a weekly EBM teaching course for an hour in the morning. Students were also required to participate in EBM lectures run by the hospital, and learn EBM concepts and skills from other conventional in-class or online teaching programs. Overall, the total number of classroom teaching hours for the FC and LB groups was the same, ranging from 30 to 36 h. During the study period, a total of 113 students enrolled in the two courses, but after propensity score matching, the FC group and LB group were each composed of 45 students, for a total of 90 students, included in the study and analysis (Fig. 1). This study was approved by the institutional review committees at Taipei Veterans General Hospital (VGHTPE) (IRB No: 2014–11-008B, 2017–01-020 AC). The researchers obtained written informed consent from the students at the beginning of the course. Other actual procedures for research methods were also implemented in accordance with the plan attached to the IRB application and the human research ethics code of the review agency.

### Self-report questionnaire

Considering that personality traits may affect students’ learning attitudes and classroom response patterns, the researchers conducted the 40-item Big Five test [20–22] on the students before the course. This scale is based on the international version of the International Big Five Mini-Marker by Thompson et al., derived from multinational background samples; it was translated into a Chinese version by Taiwanese scholars [20, 23]. The scale includes five important personality traits: extroversion, openness, neuroticism, conscientiousness, and agreeableness on a 9-point Likert scale from “strongly disagree” to “strongly agree.” This measurement tool has been used in many studies in the field of education and has repeatedly demonstrated good reliability and validity, showing its usability [20–24].

In addition, students’ personal and learning background information, including age, sex, admission route (interview, recommendation, or national examination), student loans, part-time job, and past academic performance (average grades from the first to fourth year at university) were collected using the pre-course questionnaire.

### Objective assessments of EBM learning performance

To understand learning effectiveness in the FC and LB student groups before they were exposed to the EBM courses, researchers tested the basic concepts of EBM on students taking the classes for the first time, using 20 multiple-choice questions modified from previously published literature [16]. The details of this scale are published elsewhere [16]. The pre-course test was used as a baseline assessment of the students’ EBM skills. Scores were converted into 0–10 and were used as a covariate in the comparative analysis.

The post-test, which occurred at the end of the course, included two parts: a written test and an oral test. The written test included open-ended questions, modified from the “Fresno test” used to assess the effectiveness of a comprehensive EBM curriculum in the University of California, San Francisco’s Fresno family practice residency program [25–27]. Based on clinical scenarios set by the researchers, each student responded to the following prompts: (1) identify the most appropriate research design for answering the question, (2) show the process of database searching, (3) identify important issues for determining the relevance and validity of a given research article, and (4) discuss the significance and importance of the research findings. These prompts included the “Ask,” “Acquire,” and “Appraise” aspects of EBM (scores converted to 0–10). Exam results were assessed by five experienced raters who have discussed the difficulty level of the exam questions and consistency of scoring throughout the course consensus meeting, to minimize differences in exam question depth and subjective ratings.

The oral test was implemented by grouping students based on their EBM questions, using the population, intervention, control, and outcomes (PICO) format which is of clinical interest and suitable for in-depth discussion. Multiple individuals scored their presentation and responses to inquiries. For the oral exam, aside from the analysis of “Ask,” “Acquire,” and “Appraise” from the written exam questions, the aspect of “Applying” the integrated concepts obtained from these studies was also assessed. The oral tests were rated by at least five independent raters, who had been qualified as EBM teachers and had at least 100 h of teaching experience. Oral test scoring was based on the EBM competition checklist from the National Medical Quality Award held by the Joint Commission of Taiwan. This has been validated by EBM experts and was modified by our EBM teachers to accommodate our test format. The scoring domains included “Ask: the quality and quantity of PICO,” “Acquire: the searching strategy,” “Appraise: summarizing the validity and importance of each article,” and “Apply: transforming evidence into practice.” Detailed items and scoring are shown in Supplemental Table S1). Each domain was converted into a score of 0–10 for further analysis.

### Statistical analysis

We performed the analyses using the IBM SPSS Software version 20. For the comparative analysis, all written test scores, oral test scores, and students’ past academic performance grades were normalized from 0 to 10 points. Independent samples t-test (for continuous variables) and chi-square test (for categorical variables) were used to compare differences in population and learning background, between students in the FC and LB groups.

To reduce selection bias and to effectively adjust for possible confounding factors that may affect post-course performance, PPSMA [28, 29] was used to select students in the LB group with similar background attributes for pairing with each student in the FC group. Variables included in the propensity score modeling were age, sex, university admission route, student loans, part-time job, past academic performance in the preclinical years, personality dimension scores from the Big Five, and pre-course test scores before exposure to the EBM curriculum. These variables, including pre-course ability, personal traits, learning resource and allocated time, were included in PPSMA because they might affect students’ academic achievement and students’ learning outcomes of the EBM courses. The research flowchart is shown in Fig. 1. Propensity score matching was performed using logistic regression with the “allocation group” as the dependent variable. The propensity score, the predicted probability that a particular individual is assigned to the experimental group, was derived for each participant and used to select students for the control group.

After the two groups of students were paired, the standardized mean difference (SMD) was used to check whether the distribution of variables in the two groups was balanced. The SMD was calculated by dividing the score of the experimental group minus the control group score, by the total standard deviation. SMD < 0.1 indicated a negligible difference between the two groups. This indicator was used to determine the balance between the two groups because this was a small sample. The resulting value is more rigorous than the *p*-value, so the SMD and the *p*-value are juxtaposed.

Based on the written and oral test scores of the two student groups after completing the class, an analysis of covariance (ANCOVA) was conducted to investigate the differences in post-test scores after adjusting for the covariance between the two groups. The included covariates were the same as those used in the PPSMA. Radar charts were used to visualize the performance of written and oral test results from the two groups of students.

Finally, to investigate the difference between the written and oral test scores between the two groups of students after controlling for potential confounding variables, and to analyze the interpretation ratio of the “group” variable in the model, multivariable linear regression analyses were also performed. The adjusted R square value (adjusted R^2^) was adopted to quantify the proportion of variance explained by covariates in the regression models. Statistical analyses were performed using SPSS v18 and radar charts were generated using Python v3.7. All analyses were considered statistically significant at *P* < 0.05.

## Results

### Comparisons of baseline characteristics between the flipped classroom (experiment) and traditional classroom (control) students

During the study period, 45 and 68 students were initially recruited for the FC and LB groups, respectively. The baseline characteristics of the two groups of study participants, and the pre-course test results before propensity scores were matched, are shown in Table 1.

**Table 1:** Baseline characteristics of flipped classroom medical students and traditional lecture-based classroom students before propensity score matching (*n* = 113)

Variable	LB group (*n* = 68)		FC group (*n* = 45)		*p*-value
	n/mean	%/sd	n/mean	%/sd	
Age	23.85	(1.09)	23.20	(3.17)	0.121
Sex					0.606
Male	39	(57.4%)	28	(62.2%)	
Female	29	(42.6%)	17	(37.8%)	
Admission route					0.207
Interview	33	(48.5%)	16	(35.6%)	
Recommendation	13	(19.1%)	7	(15.6%)	
Examination	22	(32.4%)	22	(48.9%)	
Student Loan					0.089
Yes	4	(5.9%)	7	(15.6%)	
No	64	(94.1%)	38	(84.4%)	
Part-time job					0.285
Yes	22	(32.4%)	19	(42.2%)	
No	46	(67.6%)	26	(57.8%)	
Personalities (Big Five Mini-markers)					
Extroversion	46.12	(10.59)	42.02	(11.77)	0.056
Openness	45.87	(5.42)	46.46	(9.29)	0.667
Neuroticism	38.61	(8.09)	39.53	(8.42)	0.564
Conscientiousness	48.60	(6.98)	49.66	(7.81)	0.451
Agreeableness	54.28	(7.05)	53.58	(7.07)	0.606
Past academic performance	4.39	(0.95)	4.83	(0.78)	0.011
Pre-course test score in the EBM class	6.03	(1.65)	7.31	(1.41)	< 0.001

*sd* Standard deviation, *LB* Lecture-based, *FC* Flipped classroom, *EBM* Evidence-based medicine

**Fig. 2:**
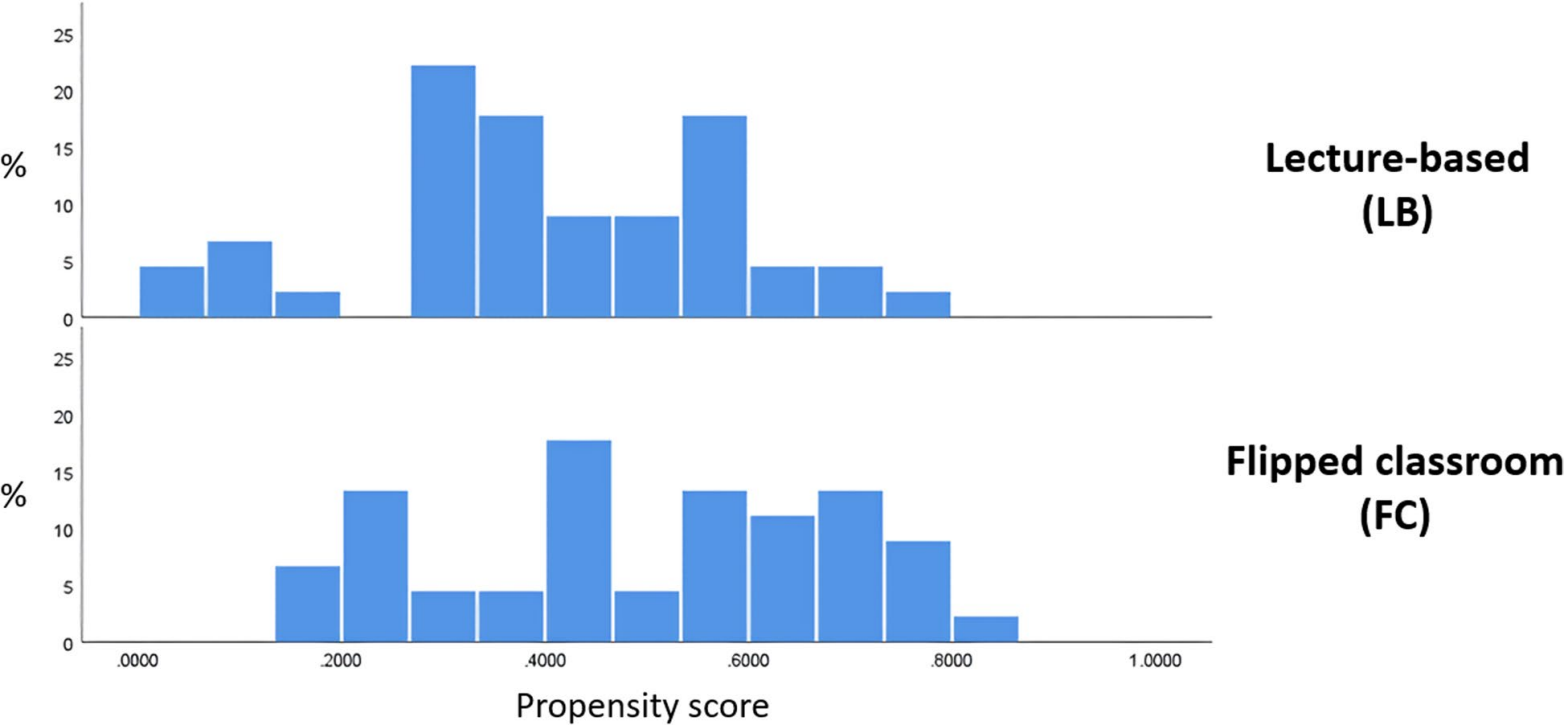
Distribution of propensity scores before matching of the two groups

Table 2 and Fig. 2 present the descriptive analysis of the average and distribution ranges of the propensity scores for the two groups. The degree of overlap and proportion of numerical ranges were used to evaluate the comparability of the two groups. Despite considerable overlap, a substantial difference in propensity scores between the two groups was observed.

**Table 2:** Distribution of propensity scores between the traditional lecture-based and flipped classroom groups before propensity score matching (*n* = 113)

		Propensity score			
Group	*n*	mean	sd	min	max
Traditional	68	0.3336040	0.18080857	0.05167	0.79113
Flipped	45	0.4958873	0.18884136	0.16619	0.81352
Total	113	0.3982301	0.19983475	0.05167	0.81352

*sd* Standard deviation

The background characteristics of the two groups of participants after 1:1 propensity score matching and the pre-course test results are shown in Table 3.

**Table 3:** Baseline characteristics of flipped classroom medical students and traditional lecture-based classroom students prospectively selected by propensity score matching (*n* = 90)

Variable	LB group (*n* = 45)		FC group (*n* = 45)		SMD	*p*-value
	n/mean	%/sd	n/mean	%/sd		
Age	23.81	(1.15)	23.20	(3.17)	0.272	0.229
Gender					0.134	0.667
Male	26	(57.8%)	28	(62.2%)		
Female	19	(42.2%)	17	(37.8%)		
Admission route					0.146	0.223
Interview	24	(53.3%)	16	(35.6%)		
Recommendation	6	(13.3%)	7	(15.6%)		
vExamination	15	(33.3%)	22	(48.9%)		
Student Loan					0.373	0.079
Yes	2	(4.4%)	7	(15.6%)		
No	43	(95.6%)	38	(84.4%)		
Part-time job					0.090	0.384
Yes	15	(33.3%)	19	(42.2%)		
No	30	(66.7%)	26	(57.8%)		
Personalities (Big Five Mini-markers)						
Extroversion	45.10	(10.46)	42.02	(11.77)	0.377	0.193
Openness	46.23	(5.48)	46.46	(9.29)	0.014	0.881
Neuroticism	37.67	(8.22)	39.53	(8.42)	0.126	0.290
Conscientiousness	49.60	(6.31)	49.66	(7.81)	0.073	0.965
Agreeableness	54.51	(6.89)	53.58	(7.07)	0.228	0.527
Past academic performance	4.67	(0.58)	4.83	(0.78)	0.412	0.266
Pre-course test score in the EBM class	6.52	(1.65)	7.31	(1.41)	0.828	0.017

*LB* Lecture-based, *FC* Flipped classroom, *EBM* Evidence-based medicine

It was found that the SMD values, including age, sex, admission route, student loans, past academic performance, pre-course scores in the EBM class, and extroversion, neuroticism, conscientiousness, and agreeableness in the personality trait scales were larger than 0.1; therefore, these variables were included in subsequent ANCOVA and multivariable linear regression models.

### The comparison of post-test learning outcomes for different EBM aspects between the FC and LB groups

Table 4 presents the post-test scores of the FC and LB groups on the written and oral tests with ANCOVA *p*-values less than 0.001. In the FC group, the scores for all aspects of the written and oral tests were all significantly higher than those of the LB group.

**Table 4:** Comparison of post-test outcomes between flipped classroom and traditional classroom medical students at the end of the EBM program^a^ (*n* = 90)

Post-course scores for the EBM program (EBM category/exam format)	LB group (*n* = 45)		FC goup (*n* = 45)		SMD	*p*-value
	mean	sd	mean	sd		
Ask/written exam	3.87	(1.13)	6.22	(1.27)	1.959	< 0.001
Acquire/written exam	3.94	(1.64)	6.40	(1.72)	1.459	< 0.001
Appraise/written exam	1.47	(1.74)	6.87	(2.20)	2.724	< 0.001
Ask/oral exam	6.12	(0.88)	7.45	(0.91)	1.487	< 0.001
Acquire/oral exam	5.63	(1.19)	7.47	(1.14)	1.577	< 0.001
Appraise/oral exam	6.87	(0.86)	7.56	(0.73)	0.867	< 0.001
Apply/oral exam	3.97	(1.24)	7.34	(0.80)	3.240	< 0.001

*sd* Standard deviation, *smd* Standardized mean difference, *LB* Lecture-based, *FC* Flipped classroom, *EBM* Evidence-based medicine

^a^Aspect “Apply” to testing from oral examinations only

**Fig. 3:**
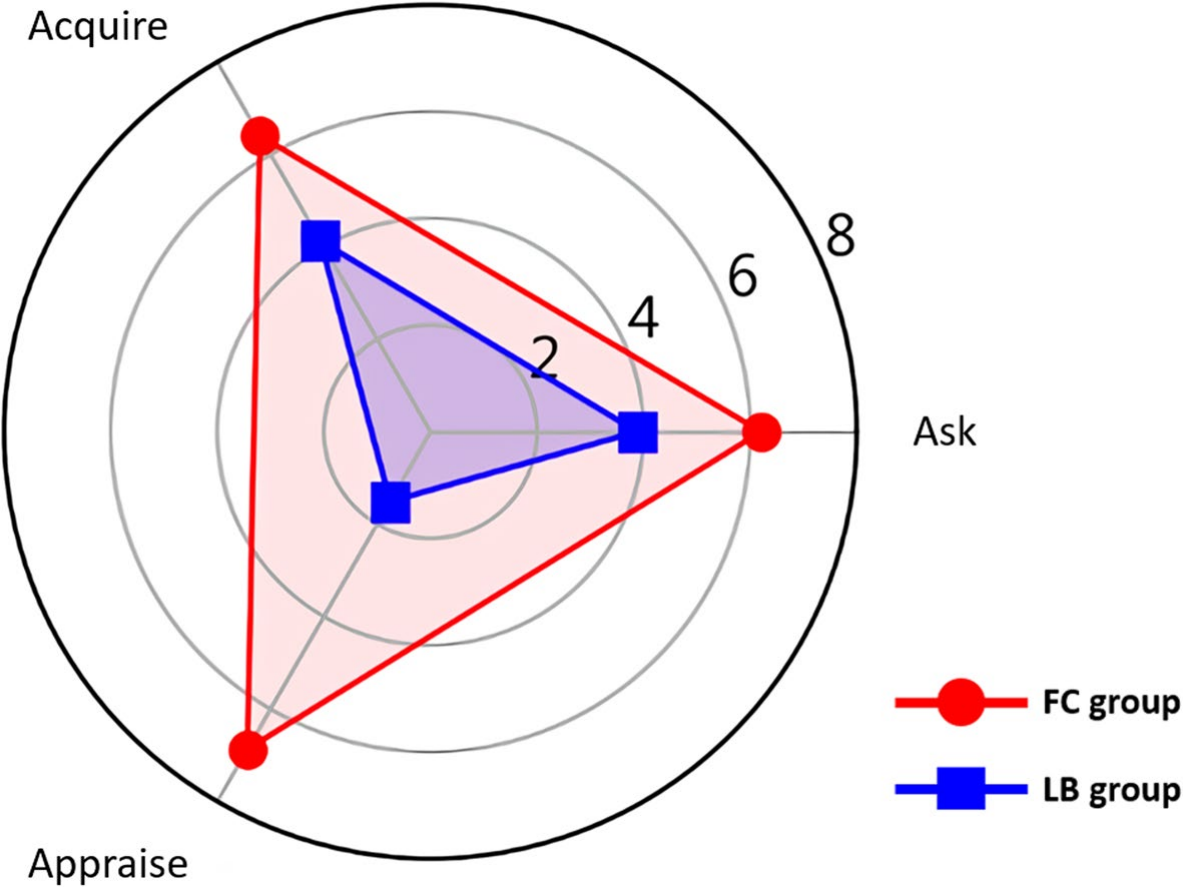
Comparison of written test scores between the two groups

**Fig. 4:**
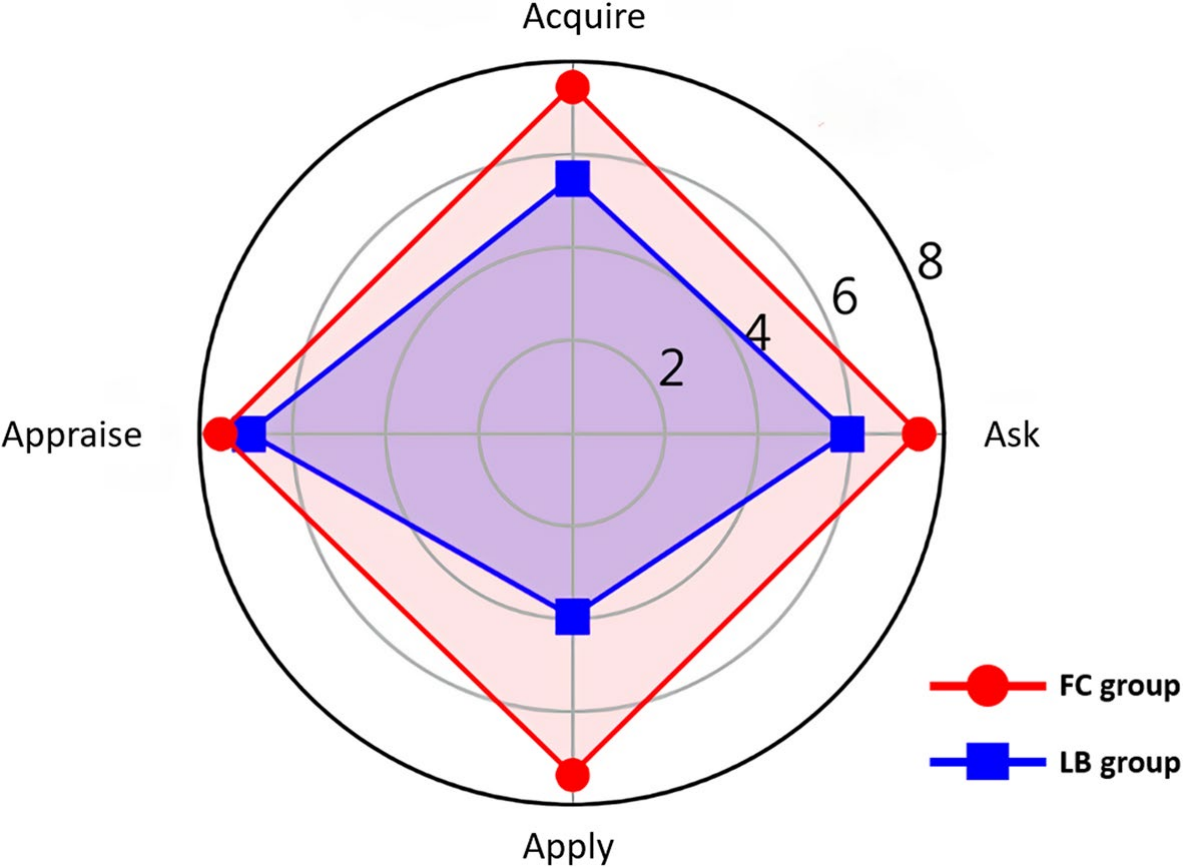
Comparison of oral test scores between the two groups

We used radar charts for further visualization of the comparisons of different EBM competencies (Figs. 3 and 4). The differences in scores for each aspect between the two groups were statistically significant. For the written test, the gap was the largest for the Appraisal aspect, while the largest difference was noted for Apply aspect of the oral test score. For these tests, the significant difference between the two groups shows the effectiveness of a FC for teaching EBM competency, especially in translating knowledge into clinical practice.

We then combined the two groups of students and performed multivariable linear regression analyses. These results are shown in Table 5. The FC group had better results than the control group for all written and oral test results. Comparing regression models with different aspects of EBM competency as the dependent variables, it was found that the adjusted R^2^ for the “allocation group” variable of the Appraise aspect of the written exam (0.66) and the Apply aspect of the oral exam (0.65) were the largest, while the Appraise (0.25) and Ask aspects of the oral exam (0.39) were the smallest. On the other hand, the average course satisfaction score of the FC and LB group were 4.69 and 4.22 points, respectively (p for between group comparison< 0.001; score ranged from 1 to 5 points, the higher the score, the more satisfied); the FC group also gave a very positive evaluation of the course in their subjective feedback.

**Table 5:** Regression coefficient for “allocation groups” in the multivariable linear regression models including EBM post-course exam scores for all students^a^ (*n* = 90)

Post-course scores for the EBM program (EBM category/exam format)	Unstandardized coefficient		Standardized coefficient	t	*p*-value	Adjusted R^2^
	B	se	β			
Ask/written exam	2.219	.265	0.649	8.377	< 0.001	0.496
Acquire/written exam	1.950	.345	0.478	5.651	< 0.001	0.400
Appraise/written exam	5.447	.415	0.839	12.419	< 0.001	0.658
Ask/oral exam	1.659	.224	0.634	7.409	< 0.001	0.387
Acquire/oral exam	1.908	.261	0.616	7.324	< 0.001	0.406
Appraise/oral exam	0.654	.171	0.360	3.815	< 0.001	0.253
Apply/oral exam	3.396	.264	0.834	12.846	< 0.001	0.647

^a^Age, gender, admission route, student loan, past academic performance, pre-course scores in the EBM class, extroversion, neuroticism, conscientiousness, and agreeableness scores from the personality trait scales with SMD > 0.1 listed in Table 3 were included as covariates. The unstandardized regression coefficient for the “group” variable refers to the control group as the reference group, and the average score difference of the experimental group relative to the control group (standardized coefficient is the average score difference of the experimental group relative to the original score of the control group after standardization)

## Discussion

This study used a rigorous PPSMA analytic framework to present empirical evidence for implementing the FC teaching model to teach EBM competency. For all aspects of EBM competency, including Ask, Acquire, Appraise, and Apply, students in the FC teaching group performed significantly better than the students in the LB group. This study also found that for higher-level competencies, such as Appraise (written exam) and Apply (oral exam) the evidence, the difference in performance between the two groups was more pronounced. These findings indicate that the FC model is a useful and efficient teaching method for all EBM competencies. In our previous experience, students could not overcome barriers to learning EBM, because they lacked practice and real-time problem solving skills. In the FC method, students have discussions with their peers through pre-class video clips, ask questions through the study platform, and resolve their doubts when sharing their answers in class. The interactive approach reduced learning gaps and helped students utilize the knowledge for clinical decisions, facilitating further utility of EBM in medical care. We believe that the FC training method helps students break out of the learning routines they previously followed, and provides more incentives for students in the positive spirit of autonomy, positivity, cooperation, inquiry, etc. that have been emphasized in the FC literature in the past [6–9]. The multi-faceted skill assessment of EBM can better reflect the effectiveness of learning attitudes beyond the knowledge-oriented written test. Moreover, our study endeavored to avoid the selection bias that often exists in non-randomized controlled studies. The matched control group made our results more convincing than most of the observational educational studies that utilized only pre- and post-study comparisons.

The current study findings on the use of the FC teaching model add to the body of evidence on teaching the tenets of EBM. A number of studies investigating different teaching methods were evaluated in one systematic review on the effectiveness of EBM teaching methods. This review concluded that the body of evidence available to guide educators on how to teach EBM to medical trainees is small, and further research is required to determine the effects of timing, content, and length of EBM courses and teaching methods [30]. However, studies included in this review were limited to randomized control trials (RCTs), but many empirical studies involving real-world teaching situations cannot be implemented using random assignment, so they were excluded. In other studies that used meta-analysis to analyze the effectiveness of FC teaching, less than 20% of the included studies were RCTs [11, 31]. Excluding non-randomized controlled studies may leave out a lot of available evidence from good quality research.

There are some reasons underlying the significant differences in the learning outcomes between the FC and LB groups. We observed the implementation process for teaching activities in the two groups and noticed that the characteristics of the curriculum may contribute to such differences. For example, in the FC group, by allowing students to engage in interactive discussions between teachers and peers after studying the pre-class materials, students may have more in-depth content knowledge, which can facilitate the discussion about the abstract concept of EBM principles. In contrast, students in the LB group simply received knowledge in the classroom, without having interactions and discussions beyond the classroom; the learning situation of students was similar to that of our EBM courses in the past before the innovative teaching was attempted [16]. Therefore, a higher degree of internalization of the knowledge and stimulating reflections from each student could not be achieved through the traditional learning method. EBM is not only a type of static knowledge, but a high-level skill that transforms knowledge into effective thinking, judgment, and decision-making [15, 32]. Curriculum design based on the FC teaching model may be more conducive to the development of EBM’s ability to apply meta-knowledge; it was also found that students’ subjective feedbacks about the course were more positive. Although it is hard to identify the exact effectiveness of FC teaching and knowledge retention, our post-classroom questionnaires and feedbacks provide us with the evidence that students are empowered to integrate EBM knowledge into their clinical curriculum.

Our study used the PPSMA method to control for the influence of multiple possible confounding factors on the evaluation of learning effectiveness in quasi-experimental and observational research, which helps enhance the confidence in the internal validity of the research results. However, there are still some limitations in this study. First, since the FC teaching is more suitable for small class teaching, this study has enrolled students from 2016 to 2018 school year, but the sample size is still limited. However, the results demonstrated that there was still sufficient statistical power. Besides, it is still possible that there may still be other unobserved variables that might bias the evaluation of the intervention effect. Theoretically, it is very difficult for the PPSMA to include all possible sources of learning performance in the assessment. However, we included as many potential confounding factors as possible in the analytic framework, including age, sex, university admission route, student loans, part-time jobs, past academic performance in the preclinical years, personality dimension scores from the Big Five, and pre-course EBM scores. Because of the lack of long-term follow-up of the present study, there is limited data regarding their long-term longitudinal effectiveness of flipped classroom. Future follow-up studies should be conducted to address this important issue.

In conclusion, the empirical evidence for teaching effectiveness demonstrated in this study provides an important reference to support the large-scale application of the FC method in EBM teaching. The FC theory involves the flipping of learning concepts, learning subjects, and educational philosophical thinking. When applied to EBM education including training in asking, searching, reviewing, and applying evidence, we believe that the application of FC teaching has the potential to help students improve their mastery of EBM and their current or future practice. The COVID-19 epidemic has accelerated the digital transformation of teaching activities and may also be an opportunity to improve the integration of FC teaching into teaching design of medical education. Furthermore, the rapid growing evidence with this new disease strengthen the need of EBM implementation for each medical student. The results of this study may help EBM educators select the most appropriate teaching method. We believe that the application of this method to other clinical education fields may also have considerable potential. With the use of PPSMA, the evaluation of learning effects can be presented with a much more rigorous approach, for data processing and analysis. The present study represents an important step for informing clinical educators of a useful educational strategy, by sharing the successful experience of implementing the FC model in pre-clinical EBM curriculum, as well as the establishment of a rigorous framework for evaluating teaching effectiveness. It is suggested that follow-up research combined with more rigorous longitudinal assessment evidence can describe the learning process of students’ clinical thinking, cognition and behavior change in more details, and also build up the empirical basis of EBM education with a sounder foundation.

## Supplementary Information

**Additional file 1: Supplemental Table S1.** Oral Test Score Checklist*.

## Data Availability

The dataset analysed during the current study is available in the Dropbox repositor, download site: https://www.dropbox.com/s/kx4de8ohb3h35po/20220112_flipped%20classroom_data_final.xlsx?dl=0
