# Supplementary material for: Implementing a flipped classroom model in an evidence-based medicine curriculum for pre-clinical medical students: evaluating learning effectiveness through prospective propensity score-matched cohorts
Source: BMC Med Educ. 2022 Mar 16;22:185. doi: 10.1186/s12909-022-03230-z (PMC8925289; doi:10.1186/s12909-022-03230-z)
Supplement: Supplementary file 1 — Additional file 1: Supplemental Table S1. Oral Test Score Checklist*. [file 12909_2022_3230_MOESM1_ESM.doc]

Supplemental Table S1. Oral Test Score Checklist*

| **Items** | | **Score** | | | | |
| --- | --- | --- | --- | --- | --- | --- |
| **Ask** | 1. The patient population and target diseases are clearly described. | 5 | 4 | 3 | 2 | 1 |
|  | 2. The intervention or other confounding variables from PICO are defined. | 5 | 4 | 3 | 2 | 1 |
|  | 3. The specific target outcomes from PICO are defined. | 5 | 4 | 3 | 2 | 1 |
| **Acquire** | 1. The correct keywords were used in searching for articles. | 5 | 4 | 3 | 2 | 1 |
|  | 2. The search strategy was clearly described. | 5 | 4 | 3 | 2 | 1 |
|  | 3. The search efficacy was enhanced through advanced search features. | 5 | 4 | 3 | 2 | 1 |
|  | 4. Articles were chosen after the search in an analytical manner. | 5 | 4 | 3 | 2 | 1 |
| **Appraise** | 1. The correct appraisal sheet was used. | 5 | 4 | 3 | 2 | 1 |
|  | 2. The “validity” of the article was thoroughly and carefully appraised. | 5 | 4 | 3 | 2 | 1 |
|  | 3. The “importance” of the article was thoroughly and carefully appraised. | 5 | 4 | 3 | 2 | 1 |
|  | 4. Evidence was correctly integrated and the article was scored. | 5 | 4 | 3 | 2 | 1 |
| **Apply** | 1. The evidence was applied to the target patient population or other similar group. | 5 | 4 | 3 | 2 | 1 |
|  | 2. The cost-effectiveness and feasibility of the health insurance system was considered. | 5 | 4 | 3 | 2 | 1 |
|  | 3. Different treatment options were offered using clinical decision strategies. | 5 | 4 | 3 | 2 | 1 |
|  | 4. The patient’s expectations, concerns, and viewpoint were included in applying the evidence. | 5 | 4 | 3 | 2 | 1 |

*A reason should be noted when the specific item score is below 2.
